# Clinical Profile and Quality of Life of Patients With Post-pulmonary Tuberculosis Sequelae Presenting to a Tertiary Care Hospital

**DOI:** 10.7759/cureus.36354

**Published:** 2023-03-19

**Authors:** Zubair Ahmad Thoker, Karan Madan, Saurabh Mittal, Pawan Tiwari, Tajamul Hussain Shah, Anant Mohan, Vijay Hadda, Randeep Guleria

**Affiliations:** 1 Pulmonary Medicine, Sher-i-Kashmir Institute of Medical Sciences Soura, Srinagar, IND; 2 Pulmonary, Critical Care and Sleep Medicine, All India Institute of Medical Sciences, New Delhi, New Delhi, IND; 3 Pulmonary, Critical Care and Sleep Medicine, School of Excellence in Pulmonary Medicine, NSCB Medical College, Jabalpur, IND

**Keywords:** sgrq, quality of life, clinical profile, sequelae, pulmonary tuberculosis

## Abstract

Background and objective

There is a dearth of studies on the clinical presentation of patients with post-pulmonary tuberculosis (PTB) sequelae and its impact on their quality of life (QoL). In light of this, we conducted this study to analyze the clinical profile and QoL in patients with post-PTB sequelae.

Methods

Patients with a history of treated PTB and evidence of radiological damage were enrolled prospectively from November 2018 till June 2020 to study their clinical profile and QoL as per the eligibility criteria. A detailed clinical history was taken along with posteroanterior-view chest X-rays and CT scans of the thorax with bronchial angiography in patients with hemoptysis. QoL was assessed using the Hindi version of St. George's Respiratory Questionnaire (SGRQ) for which permission was obtained from the St. George’s University of London. SGRQ scores were calculated using score calculation algorithms (Microsoft Excel-based) and missing data imputation as recommended by its developer.

Results

A total of 174 patients were included in the analysis. The analyzed population was relatively younger (mean age: 44.27 years) with BMIs leaning toward the lower side of normal (median: 19.6 kg/m²); the majority of the patients were males (59%) and non-smokers (77%). PTB had been diagnosed clinico-radiologically in the majority (68%) of patients with non-compliance to antitubercular treatment (ATT) being reported by only 9% of patients. Multiple courses of ATT were received by about one-third of patients, mainly on a clinico-radiologic basis. Systemic hypertension (HTN) (11%) and diabetes mellitus (DM) (9%) were the most common comorbidities. The most common symptom complex found was cough, expectoration, and dyspnea (n=102, 59%). At least one incidence of massive hemoptysis was reported by 20% of patients. Bronchial artery embolization (BAE) was performed for moderate to massive hemoptysis in 26% of patients with a success rate of >90%. One-fifth of the study participants required hospitalization for exacerbation of respiratory symptoms with more than half of these (59%) requiring ventilatory support. Health-related QoL was significantly impaired as reflected by a median SGRQ total score of 45.53. The most affected domain of QoL was the activity domain (mean score: 45.47). Females had worse QoL as compared to males (p=0.0062), and so did underweight patients (p=0.048). The prolonged duration of the illness also significantly impaired the QoL (p<0.001, r=0.313).

Conclusion

The sequelae of PTB are under-recognized even among physicians and are frequently misdiagnosed as active PTB. The QoL is more severely affected due to residual damage. This study highlights the clinical profile of this patient population and underscores the need to recognize post-PTB sequelae as a separate entity. An important remedy to mitigate its long-term consequences is its inclusion and recognition in national and international TB guidelines to facilitate its early identification and promote further research to address its evidence-based management.

## Introduction

Pulmonary tuberculosis (PTB) can lead to irreversible lung damage, manifesting as scarring, fibrosis, cavitation, or other types of damage on radiological images. This damage can lead to loss of lung function, long-term respiratory symptoms, and eventually, chronic respiratory disease, including chronic obstructive pulmonary disease (COPD), bronchiectasis, and aspergillosis [1-5]. However, the current WHO-recommended TB registries only focus on mortality and morbidity during TB treatment and, as a result, patients are not followed up beyond the cure of the disease [6]. Out of hundreds of international TB guidelines, only a few mention TB sequelae, and none describe how to identify or manage the condition. This could be attributed to the lack of studies focusing on post-PTB sequelae.

Although smoking remains the most crucial risk factor for COPD, a considerable burden of the disease in developing countries cannot be explained by smoking alone. TB and other non-smoking-related risk factors of COPD have been well described [7]. The relationship between TB and COPD in non-smokers is supported by data from the Burden of Obstructive Lung Disease (BOLD) and Proyecto Latinoamericano de Investigación en Obstrucción Pulmonar (PLATINO) studies, which performed sub-group analyses and found even more robust associations between TB and COPD in people who were never-smokers [8,9]. A systematic review and meta-analysis found that the association between prior history of TB and the presence of COPD was strongest in never-smokers and younger people (<40 years of age) [10].

As the patients of PTB are rarely followed up upon completion of the treatment, there is scarce data on their sociodemographic parameters and symptomatology of post-PTB sequelae. It is unknown how physicians usually approach such patients. It is not uncommon to find such patients being managed as active PTB cases on clinico-radiologic grounds, particularly by primary care practitioners due to a lack of awareness about post-PTB sequelae. The presentation of respiratory failure and management in such patients has rarely been documented in the literature. The effect of impaired pulmonary function on health-related quality of life (QoL) has been studied sparingly. A few studies have observed a correlation between spirometry measures and components of St. George's Respiratory Questionnaire (SGRQ) scores as well as overall [11,12].

Globally, good quality data on the clinical profile and QoL of patients with post-PTB sequelae are sparse, particularly from India where TB is endemic. Hence, this study was planned to perform a comprehensive evaluation of the clinical profile and QoL of patients with post-PTB sequelae.

## Materials and methods

This was an observational cross-sectional study involving patients attending the Department of Pulmonary Medicine, All India Institute of Medical Sciences (AIIMS), New Delhi between November 2018 and June 2020 who were selected based on the eligibility criteria. All patients who had microbiologically or clinico-radiologically proven and treated PTB as per national guidelines with radiological evidence of sequelae were defined as post-PTB sequelae cases. They would undergo a detailed evaluation, including clinical history, posteroanterior-view chest X-rays, and CT scans of the thorax with bronchial angiography in patients with hemoptysis. All patients would undergo sputum for acid-fast bacillus (AFB) and cartridge-based nucleic acid amplification test (CB-NAAT) to rule out active PTB. As per the clinico-radiological assessment, bronchoalveolar lavage (BAL) was done to rule out active TB, wherever indicated. The QoL was assessed using the Hindi version of SGRQ for which permission was obtained from the St. George’s University of London. SGRQ scores were calculated using score calculation algorithms (Microsoft Excel-based) and missing data imputation as recommended by its developer (P.W. Jones, St Georges Hospital Medical School, London, UK). Patients with active PTB, prior diagnosis of asthma or COPD, bronchiectasis due to non-tubercular causes, interstitial lung disease, pneumoconiosis (like silicosis, coal miner’s pneumoconiosis, and asbestosis), and other causes of pulmonary fibrosis, history of thoracotomy, and patients unwilling to give consent were excluded from the study.

Data management and statistical analysis

Data entry was done as per the designed proforma. Study data were collected and managed using REDCap electronic data capture tools hosted at AIIMS, New Delhi. Data management and analysis software Stata, version 14.2 was used for analysis. We used mean and standard deviation (SD) to represent the continuous variables when the variable followed a normal distribution. In the case of non-normal continuous variables, we used the median and interquartile range (IQR). The categorical variables were represented as frequencies with percentages. Categorical variables were compared between the groups by Chi-square/Fisher's exact test. Wilcoxon rank-sum test was applied when the continuous variable did not follow a normal distribution. A p-value <0.05 was considered statistically significant.

## Results

**Figure 1:**
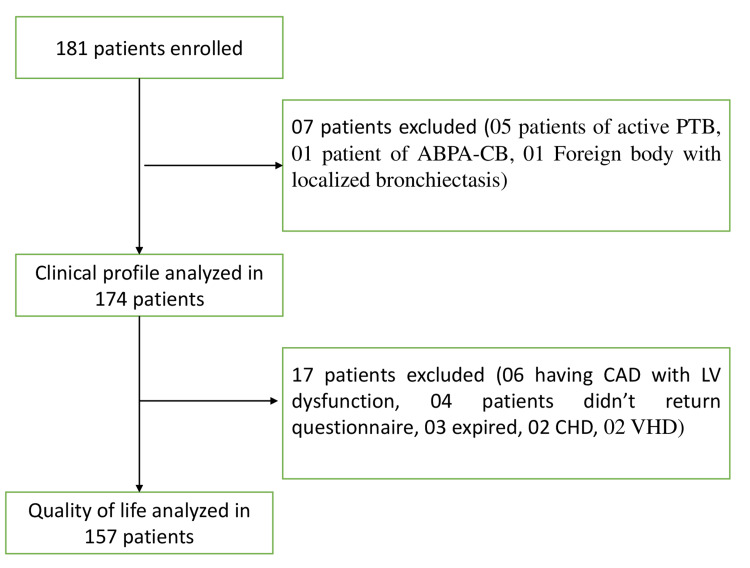
Flowchart depicting the patient selection ABPA-CB: allergic bronchopulmonary aspergillosis-central bronchiectasis; CAD: coronary artery disease; CHD: congenital heart defect; LV: left ventricle; PTB: pulmonary tuberculosis; VHD: valvular heart disease

A total of 181 patients were enrolled in the study. Of these, 174 were included in the analysis (Figure 1). We excluded five patients who were found to have a recurrence of active PTB, one patient who had allergic bronchopulmonary aspergillosis-central bronchiectasis (ABPA-CB), and one patient who had suspected intrabronchial foreign body with left lower lobe bronchiectasis.

The majority (83.33%) of the affected patients were in the age group of 20-60 years with a mean age at enrollment of 44.27 years; 71 (40.80%) participants were females. The median (IQR) BMI was 19.6 (17.55-22.9) kg/m^2^. Of the 174 patients, 59 (33.90%) were underweight (BMI <18.5 kg/m^2^), and 24 (13.80%) were overweight (BMI ≥25 kg/m^2^). Smoking history was given by 40 (22.98%) patients with only five (12.5%) of them being cigarette smokers. Of the 71 female patients included in the study, 39 (54.92%) had experienced biomass fuel exposure in their lifetime. The baseline characteristics of the analyzed participants are presented in Tables 1-2.

**Table 1:** Baseline characteristics - 1 (n=174) IQR: interquartile range; SD: standard deviation

Characteristics			
Age in years, mean ± SD			44.27 ± 14.63
Gender, n (%)	Male		103 (59.2)
	Female		71 (40.80)
BMI, Kg/m^2^, median (IQR)	19.6 (17.55-22.9)		
BMI, Kg/m^2^, n (%)	Normal		91 (52.30)
	Underweight		59 (33.90)
	Overweight		24 (13.80)
Smoking status, n (%)	Smokers: 40 (22.98)	Bidi	35 (87.5)
		Cigarette	5 (12.5)
		Others	0
	Non-smokers		134 (77.01)
Biomass exposure in females, n (%)			39 (54.92%)

**Table 2:** Baseline characteristics - 2 (n=174) ATT: antitubercular treatment; CB-NAAT: cartridge-based nucleic acid amplification test; IQR: interquartile range; MDR: multidrug-resistant

Characteristics			
TB diagnosis, n (%)	Clinico-radiologic		118 (67.81)
	Microbiologic: 56 (32.18)	Smear +ve	48
		CB-NAAT +ve	6
		Smear and culture +ve	1
		Smear and CB-NAAT +ve	1
The duration between the first ATT completion and the appearance of symptoms, months, median (IQR)			36 (1-120)
Duration of ATT received per patient, months, median (IQR)			9 (6-12)
No. of ATT courses received, n (%)	1		118 (67.82)
	2		41 (23.56)
	3		11 (6.32)
	4		2 (1.15)
	5		1 (0.57)
	9		1 (0.57)
Regimen received, n (%)	CAT I		166 (95.40)
	CAT II		5 (2.88)
	MDR regimen		3 (1.72)
	CAT I and CAT II		44 (25.28)
Compliance with ATT, n (%)	Compliant		159 (91.38)
	Non-compliant		15 (8.62)
Any comorbidity, n (%)	Present		46 (26.44)
	Absent		128 (73.56)

The diagnosis of PTB was on a clinico-radiologic basis in the majority of the participants (67.8%) with a median (IQR) duration between the first antitubercular treatment (ATT) completion and the appearance of symptoms being 36 (1-120) months. The median (IQR) duration of ATT received per patient was nine (6-12) months; 15 (9%) patients reported a history of ≥3 courses of ATT intake whereas 41 (23.56%) participants reported an intake of two courses of ATT. The most commonly used initial regimen at the time of diagnosis of TB was category I in 166 (95%) participants; 44 (25%) patients had been prescribed category I followed by category II treatment after varying intervals of time. Non-compliance to the first ATT intake was reported by only 15 (9%) participants.

Common comorbidities in the analyzed participants were hypertension (HTN) (11%), type 2 diabetes mellitus (DM) (09%), and coronary artery disease (CAD) (5%); 11 (06%) participants had ≥2 comorbidities with HTN and CAD being the most common combination (3.4%). Table 3 depicts the associated comorbidities in the participants.

**Table 3:** Frequency of comorbidities *CHD: 2; VHD: 2; PSVT: 1; hyperthyroidism: 2; CKD: 1; NCC: 1; HIV: 1; primary infertility: 1; RA: 1; aplastic anemia: 1; squamous cell carcinoma vallecula: 1 CAD: coronary artery disease; CHD: congenital heart defect; CKD: chronic kidney disease; CLD: chronic liver disease; DM: diabetes mellitus; HIV: human immunodeficiency virus; HTN: hypertension; NCC: neurocysticercosis; PSVT: paroxysmal supraventricular tachycardia; RA: rheumatoid arthritis; VHD: valvular heart disease

Comorbidity	N (%) (n=174)
HTN	19 (10.91)
DM	15 (08.62)
CAD	8 (4.59)
Hypothyroidism	5 (2.87)
CLD	4 (2.29)
Others*	14 (8.04)
Multiple comorbidities	11 (6.32)
HTN + CAD	6 (3.44)
HTN + DM	5 (2.87)
HTN + DM + CAD	3 (1.72)

The median (IQR) duration of symptoms on presentation to us was 48 (12-84) months; the symptom complex of cough, expectoration, and dyspnea was reported by 102 (59%) participants; the symptom complex of cough, expectoration, dyspnea, and hemoptysis was present in 61 (35%) participants (Table 3). Modified Medical Research Council (mMRC) grade 2 was the most common grade of dyspnea present (n=71, 53%) in patients; massive hemoptysis of at least one episode was present in 34 (20%) patients; two (1%) patients also had hoarseness of voice. Seasonal worsening of symptoms and wheezing were reported by 22% and 17% of participants respectively. Dull aching chest pain was present in 12% of patients. Table 4 summarizes the symptomatic presentations among the study participants.

**Table 4:** Symptoms at the time of enrollment ATT: antitubercular treatment; IQR: interquartile range; mMRC: Modified Medical Research Council

Clinical characteristics		Values (n=174)
Cough, n (%)		161 (92.52)
Expectoration, n (%)		119 (68.39)
Dyspnea, n (%)		135 (77.58)
mMRC grade of dyspnea, n (%)	0	3 (2.22)
	1	35 (25.92)
	2	71 (52.59)
	3	23 (17.03)
	4	3 (2.22)
Hemoptysis, n (%)		108 (62.06)
Massive hemoptysis, n (%)		34 (19.54)
Chest pain, n (%)		21 (12.06)
Wheezing, n (%)		29 (16.66)
Seasonal worsening of symptoms, n (%)		39 (22.41)
Hoarseness of voice, n (%)		2 (1.14)
Cough + expectoration + dyspnea, n (%)		102 (58.62)
Duration of symptoms, months, median (IQR)		48 (12-84)
Persistence of symptoms after completion of ATT, n (%)		56 (32)

Among the treatments prescribed for dyspneic patients, the combination of long-acting beta-agonist (LABA) + inhaled corticosteroid (ICS) and long-acting muscarinic antagonist (LAMA) was received by 75 (56%) patients; LABA + LAMA combination was administered to 10 (07%) participants while LAMA alone to 17 (13%) participants. Symptomatic relief of dyspnea with inhalers was reported by 126 out of 135 (93%) dyspneic patients. Bronchial artery embolization (BAE) was performed in 46 (26.4%) participants, resulting in the control of hemoptysis in 42 (91%) patients. Hospitalization for exacerbation of respiratory symptoms due to post-tubercular chronic lung disease was reported in 34 (20%) patients with 20 (59%) of them requiring mechanical ventilatory support during exacerbation. Vaccination against pneumococcus and influenza, at least once, was reported by 21 (12%) participants. The treatment profile is presented in Table 5.

**Table 5:** Treatment profile BAE: bronchial artery embolization; ICS: inhaled corticosteroid; IMV: invasive mechanical ventilation; LABA: long-acting beta-agonist; LAMA: long-acting muscarinic antagonist; NIV: noninvasive ventilation SABA: short-acting beta-agonist; SAMA: short-acting muscarinic-antagonist

Treatment			N (%) (n=174)	
Patients on inhalers			135 (77.58)	
Type of Inhalers (n=135)		LABA + ICS + LAMA	75 (55.55)	
		LABA + ICS	24 (17.77)	
		LAMA	17 (12.59)	
		LAMA + LABA	10 (7.40)	
		SABA + SAMA	9 (6.67)	
Symptomatic response to inhalers (n=135)		Present	126 (93.33)	
		Absent	9 (6.67)	
BAE	46 (26.43)	Hemoptysis controlled	42 (91.30%)	
		Hemoptysis uncontrolled	4 (8.70%) (2 underwent lobectomy; 1 awaiting pneumonectomy; 1 expired)	
Hospitalization for exacerbation of symptoms	34 (19.54)	Mechanical ventilation during exacerbation	20 (58.82%)	
			IMV	NIV
			9	11
Vaccination (pneumococcus and influenza)			21 (12.06)	

Quality of life (QoL)

Of the 174 patients, QoL was assessed in 157 patients (90%). It could not be assessed in three patients who expired while on invasive ventilation for respiratory failure, six patients due to CAD with left ventricular (LV) dysfunction, two patients due to congenital heart disease (CHD), and two patients due to valvular heart disease (VHD); four patients did not return the questionnaire (Figure 1). The median (IQR) total score was 45.53 (24.65-55.65). Table 6 shows the various components of SGRQ.

**Table 6:** Results of the various components of SGRQ (n=157) IQR: interquartile range; SD: standard deviation SGRQ: St. George's Respiratory Questionnaire

Parameter	Mean ± SD	Median (IQR)
Symptoms score	50.20 ± 20.35	
Activity score		47.69 (29.49-60.26)
Impacts score		42.29 (16.42-57.85)
Total score		45.53 (24.65-55.65)

**Figure 2:**
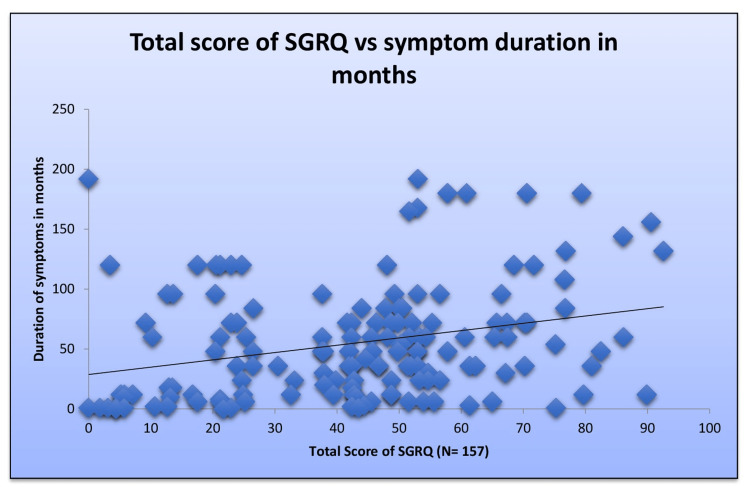
Total SGRQ score versus duration of symptoms in months Spearman correlation: r-value: 0.313; p<0.001 SGRQ: St. George's Respiratory Questionnaire

The QoL depicted by the mean total score of SGRQ was analyzed in terms of gender, smoking status, exposure to biomass, and BMI (Tables 7, 8, 9, 10 respectively); females had a significantly worse QoL as compared to males (p=0.0062); The was a significant difference in QoL between patients with BMI <18.5 Kg/m^2^ and those with BMI ≥18.5 Kg/m^2^ (p=0.048) with undernourished individuals having a worse QoL. A significant positive correlation was found between the total score of SGRQ and the duration of being symptomatic, implying that a more prolonged duration of illness is associated with a worse QoL for the patients (Figure 2).

**Table 7:** Quality of life in terms of gender (n=157) IQR: interquartile range; SGRQ: St. George's Respiratory Questionnaire

Variable	Gender		P-value
Total SGRQ score, median (IQR)	Males	Females	0.0062
	42.43 (21.18-54.32)	50.04 (41.9-60.86)	

**Table 8:** Quality of life in terms of smoking status (n=157) SD: standard deviation; SGRQ: St. George's Respiratory Questionnaire

Variable	Smoking status		P-value
Total SGRQ score, mean ± SD	Smokers	Non-smokers	0.308
	39.90 ± 21.56	43.91 ± 22.67	

**Table 9:** Quality of life in terms of biomass exposure (n=157) SD: standard deviation; SGRQ: St. George's Respiratory Questionnaire

Variable	Biomass exposure		P-value
Total SGRQ score, mean ± SD	Present	Absent	0.384
	46.75 ± 20.32	42.02 ± 22.97	

**Table 10:** Quality of life in terms of BMI (n=157) BMI: body mass index; SD: standard deviation; SGRQ: St. George's Respiratory Questionnaire

Variable	BMI, Kg/m^2^		P-value
Total SGRQ score, mean ± SD	<18.5	≥18.5	0.048
	48.72 ± 21.84	40.17 ± 22.28	

## Discussion

This was a tertiary care hospital-based cross-sectional study to assess the clinical profile and estimate the long-term effects of treated PTB on the QoL of patients. Despite having no evidence of active PTB, patients treated for PTB had substantial impairment in QoL. In our study, the affected population predominantly belonged to the younger, economically productive age group (mean age: 44 years) with 59% of the participants being males. The median BMI was 19.6 kg/m^2^ with about one-third (33.90%) of the participants being underweight; smoking history was given by 23% of patients with 35 (87.5%) of them being bidi smokers, thus reflecting the lower socioeconomic status of the affected population. Among female participants in the study, more than half (54.92%) had biomass fuel exposure for varying duration. The preponderance of post-PTB sequelae in the younger age group is in line with other studies [8,13]. The predominance of males was also reported in the studies by Akkara et al. and Radovic et al., where males accounted for 74 and 62.5% of cases respectively [13,14]. Also, in the study by Mbatchou Ngahane et al., 146 (54%) of the study participants were males; 242 (90%) of their cases were never-smokers; however, only eight (3%) patients in their study were underweight with 63% of patients having a normal BMI [15].

HTN (11%) and DM (9%) were the two most common comorbidities in our study participants followed by CAD (5%). Multiple comorbidities were present in 11 (6%) participants with the combination of HTN and CAD being the most common combination found. Mbatchou Ngahane et al. in their study reported HTN and DM in 12 (4.5%) and eight (3%) cases respectively [15].

Multiple courses (≥2) of ATT were given to 56 patients (32%) for treating suspected relapse of PTB on a clinic-radiologic basis without microbiologic confirmation, possibly due to the failure of recognizing healed PTB sequelae as a separate disease entity, thereby unnecessarily increasing potential of drug-related adverse events with no mitigation of morbidity in patients, for which the patient had presented to the physician. Van Kampen et al. also highlighted this problem of misdiagnosing post-PTB sequelae as active PTB in resource-limited settings [16]. This is particularly relevant in cases of chronic cavitary pulmonary aspergillosis (CCPA), which has clinico-radiologic similarity with PTB, and which, if left untreated, can progress to chronic fibrosing pulmonary aspergillosis (CFPA) with permanent loss of lung function. The median interval between the first ATT completion and the appearance of symptoms attributable to post-PTB sequelae was 36 months (IQR: 1-120); the median duration of ATT received per patient was nine months (IQR: 6-12).

The majority of the patients reported being compliant (91%) to ATT when they were diagnosed with PTB. Persistence of symptoms after the completion of ATT was reported by 56 patients (32%) in our study. Other studies have shown that PTB patients who have completed treatment continue to have respiratory symptoms at the end of treatment (30-47%), after one year of treatment (40%), and after two and a half years of treatment completion (15.9%) [17,18]. Banu Rekha et al. also documented the persistence of symptoms in 58 (29%) of their patients [11]. Another study has reported 53.62% of patients developing respiratory symptoms >72 months after the completion of their ATT [19].

In our study, the median duration of symptoms at the time of enrollment was 48 (IQR: 12-84) months (Table 3). The most common symptoms were cough (93%), dyspnea (78%) (especially grade 2), expectoration (68%), and hemoptysis (62%). Massive hemoptysis of at least one episode was reported by 20% of patients. The symptom complex of cough, expectoration, and dyspnea was reported in 102 (59%) participants; the symptom complex of cough, expectoration, dyspnea, and hemoptysis was present in 61 (35%) participants.

Akkara et al. [13] in their study have documented that most of the patients (85%) presented with respiratory complaints such as cough, expectoration, or breathlessness after a mean duration of 8.9 months following the cure of TB; hemoptysis was present in 12% of the participants. Singla et al. in their study on sequelae in 46 patients with multidrug-resistant (MDR) PTB, after completion of two years of treatment, found dyspnea to be the most common residual symptom (87%) with mMRC grade 2 dyspnea present in 65% of cases [20]. In another study, cough (94%) was also a dominant symptom followed by chest pain (63%) and expectoration (51%) with dyspnea and hemoptysis reported in 34% and 28% of cases respectively [15].

In our study, 135 (77.58%) post-PTB sequelae patients with dyspnea were prescribed inhalers with most of them reporting subjective symptomatic relief. Among prescribed inhalers, the combination of LABA + ICS and LAMA was received by 75 (56%) patients, LABA + LAMA combination by 10 (07%) participants, and LAMA alone by 17 (13%) participants. Symptomatic relief of dyspnea with inhalers was reported by 126 (93%) patients. Although there are no studies or guidelines regarding the management of dyspnea in post-PTB sequelae, the prescription of inhalers to such patients at one of the top-most tertiary care referral centers in India by experienced and authoritative pulmonary physicians cannot be underrated. Nonetheless, well-designed prospective studies are still needed to identify the best option for the management of dyspnea in such patients. Hospitalization for exacerbation of respiratory symptoms due to post-PTB sequelae was found in 34 (20%) patients with 20 (59%) of them requiring mechanical ventilatory support during exacerbation, which is quite high. This reflects a common occurrence of significant permanent loss of lung function. We could not find any studies in the literature regarding the prevalence of hospitalization for exacerbations of respiratory symptoms and the requirement for ventilatory support in such patients. As recurrent and massive hemoptysis is common in post-PTB sequelae, BAE was performed in 46 (26.4%) participants, resulting in the control of hemoptysis in 42 (91%) patients. Panda et al. in their systematic review found post-PTB sequelae as one of two the most common indications of BAE for the control of hemoptysis [21].

QoL was assessed using the Hindi version of SGRQ validated in the Indian population [22]. The median (IQR) total score was 45.53 (24.65-55.65), reflecting significant impairment in health-related QoL. We did not find any effect on QoL due to smoking or biomass exposure; however, patients who had BMI <18.5 kg/m^2^ had significantly worse QoL compared to patients with BMI ≥18.5 kg/m^2^. This emphasizes the need to improve the nutritional status of patients suffering from post-PTB sequelae. A significant positive correlation was found between the total SGRQ score and the duration of being symptomatic, implying that a more prolonged duration of illness is associated with worse QoL in patients. This reflects the need to recognize the entity at the earliest. Pasipanodya et al. [23], in their study of 106 patients with post-PTB sequelae, did not find any effect of smoking on QoL. We also did not find any effect of smoking on QoL. However, another study by Banu Rekha et al. did find a significant effect of smoking on QoL in their patients with smokers faring worse [11]. They also found significantly higher total scores of SGRQ in females as compared to males, reflecting a worse QoL, which is in line with our study [11]. Among the three components of SGRQ, the score for the impact component was lower than that for the symptom and activity components in our study, which is in line with earlier SGRQ-based studies in treated PTB patients [11]. While evaluating SGRQ in our study population, we noted some of its limitations. Hemoptysis is not mentioned in SGRQ, which was present in 108 (62%) patients in our study and was sometimes the dominant symptom, and hence its effects on QoL could not be evaluated. The age group affected was younger (mean age: 44.27 ± 14.63 years) and, as a result, the effect on impact score and activity score components of SGRQ may not be reflective of the actual effect as young people are generally more healthy and active. A dull aching chest pain (12%), which was as common as wheezing (17%) in our study participants, is not mentioned in the SGRQ score, and hence its impact on QoL could not be assessed.

The strengths of our study include its location, which is one of the TB-endemic regions. Moreover, our study focused on the clinical presentation of the disease in detail as well as the approach by clinicians, which no other study has reported to date to the best of our knowledge. The study also assessed the impact on QoL, which has received scarce attention in the literature so far. The main limitation of the study is that it was hospital-based rather than community-based, and hence the extrapolation of its data can be misleading given the burden of PTB. Another limitation of the study is that the majority of our patients had their PTB diagnosed on a clinico-radiologic basis instead of having a microbiologic diagnosis that is confirmatory.

## Conclusions

The sequelae of healed PTB are under-recognized even among physicians, with consequent misdiagnosis as active PTB, which leads to erroneous management involving repeated courses of ATT, thereby predisposing patients to drug-induced toxicity. This increases morbidity and mortality, which can be further aggravated by the undetected progression of post-PTB sequelae. The QoL is also affected due to residual damage. This study highlights the clinical profile of these patients and underscores the need to recognize post-PTB sequelae as a separate entity. The best way to mitigate its long-term consequences is by inclusion and recognition of post-PTB sequelae in national and international TB guidelines to facilitate its early identification and promote further research to address its management.
